# N-Acetylcysteine Slows Down Cardiac Pathological Remodeling by Inhibiting Cardiac Fibroblast Proliferation and Collagen Synthesis

**DOI:** 10.1155/2021/3625662

**Published:** 2021-11-26

**Authors:** Jin Zhou, Jing Xu, Shan Sun, Mengyuan Guo, Peng Li, Aijuan Cheng

**Affiliations:** Department of Cardiology, Tianjin Chest Hospital, Tianjin, China

## Abstract

**Objective:**

By observing the effect of N-acetylcysteine (NAC) on the proliferation and collagen synthesis of rat cardiac fibroblasts (CFs) to explore the effect of NAC on cardiac remodeling (CR).

**Methods:**

*In vivo*, first, the Sprague Dawley (SD) rat myocardial hypertrophy model was constructed, and the effect of NAC on cardiac structure and function was detected by echocardiography, serological testing, and Masson staining. Western blotting (WB) and quantitative real-time polymerase chain reaction (qRT-PCR) were used to detect the expression level of antioxidant enzymes, and flow cytometry was used to detect the intracellular reactive oxygen species (ROS) content. *In vitro*, 3-(4,5-dimethylthiazol-2-yl)-2,5-diphenyl tetrazolium bromide (MTT) assay and 5-ethynyl-2′-deoxyuridine (EdU) staining were used to detect cell proliferation, and the expression level of the NF-*κ*B signaling pathway was detected.

**Results:**

Compared with the control group, the model group had disordered cardiac structure, reduced cardiac function, and obvious oxidative stress (OS) response. However, after NAC treatment, it could obviously improve the rat cardiac structure and cardiac function and alleviate redox imbalance and cardiology remodeling. At the same time, NAC can inhibit the activation of the NF-*κ*B signaling pathway and reduce the proliferation level of CFs and the amount of ^3^H proline incorporated.

**Conclusions:**

NAC can inhibit AngII-induced CF proliferation and collagen synthesis through the NF-*κ*B signaling pathway, alleviate the OS response of myocardial tissue, inhibit the fibrosis of myocardial tissue, and thus slow down the pathological remodeling of the heart.

## 1. Introduction

Heart failure (HF) is the terminal stage of various heart diseases, and CR is the key pathological link of HF, which is based on myocardial hypertrophy (MH) and myocardial fibrosis (MF) [1]. In the early stage of the disease, MH is an adaptive structural change of the heart in order to maintain sufficient output function in the case of increased postload or volume load caused by diseases such as hypertension. During the compensatory phase of the heart, physiological changes also include an increase in the volume of myocardial cells, an increase in the weight of the heart and left ventricle, and an increase in the interventricular septum and the thickness of the left ventricular wall. However, when the heart is under a high-load environment for a long time, MH will eventually lead to a decline in ventricular function or even HF [2]. The transition from compensatory MH to decompensation or HF is a very complicated pathophysiological process. One of its main characteristics involves fibrous tissue hyperplasia, which is the occurrence of MF. In the process of pathological MH, MF will eventually lead to the attenuation of myocardial functional tissue function, ventricular expansion, or tissue sclerosis [3]. Therefore, some studies have pointed out that inhibiting MF is a promising therapeutic direction to prevent the heart from changing from compensatory MH to decompensation or HF. In addition, under various pathological conditions, the heart undergoes remodeling characterized by MF, and CR is one of the pathophysiological foundations of HF. CFs are the main effector cells of MF, and their proliferation and collagen synthesis are important features of MF [4]. Therefore, inhibiting CF proliferation and collagen production is of great value in reversing CR.

NAC is an acetyl compound of L-cysteine, and its molecular structure contains active sulfhydryl groups. It is a strong antioxidant, which interferes with the generation of free radicals, scavenges the generated free radicals, and regulates the metabolic activity of cells [5]. In addition, NAC can also regulate gene expression and signal transduction systems, exert antiangiogenesis, inhibit the development of malignant tumors, and inhibit the generation and metastasis of new organisms, and NAC is widely used in clinical practice of respiratory and nervous systems and AIDS [6, 7]. Recently, the role of NAC in the cardiovascular system has also received wide attention from research evidence. It has been found that NAC can effectively inhibit myocardial cell apoptosis caused by ischemia-reperfusion injury (IRI) and improve cardiac function [8]. In addition, some studies have shown that NAC can play an antifibrotic role in the lungs [9], but there are few studies on the effect of NAC on cardiac fibrosis. Therefore, this study explored the effect of NAC on CFs in rats to explore their effects on CR.

The nuclear transcription factor is a type of protein that has the function of binding to a fixed nucleotide sequence in certain promoter regions to initiate gene transcription. NF-*κ*B is one of its important proteins, which can be found in a variety of cells, such as vascular endothelial cells, CFs, and cardiomyocytes, and is involved in the gene regulation of a variety of physiological and pathological processes such as inflammation, immunity, and cell proliferation and apoptosis [10–12]. In general, NF-*κ*B binds to its endogenous inhibitory protein (I*κ*B) in the homologous or heterodimer form to form a trimer, which exists in the cytoplasm and is inactivated. Once the I*κ*B is phosphorylated and dissociated from the NF-*κ*B/I*κ*B complex, the dimer and the inhibitory protein are dissociated and transferred to the nucleus, where they bind to *κ*B sites on DNA and participate in the transcription of multiple genes [13]. At present, a large number of studies have confirmed that the NF-*κ*B pathway is associated with cardiovascular disease. Therefore, we speculate that NAC may slow down MF, and MH may be regulated by the NF-*κ*B signaling pathway.

## 2. Materials and Methods

### 2.1. Laboratory Animal

Thirty clean four-month-old male rats, weighing 220-250 g, provided by Tianjin Chest Hospital Animal Center, were housed in cages, with room temperature 18-22°C, relative humidity 45-65%, and light and dark alternating for 12 hours. After 7 days of adaptive feeding, the rats were randomly divided into 3 groups. This study was approved by the Animal Ethics Committee of Tianjin Chest Hospital Animal Center.

### 2.2. Animal Model Construction

After the rats were anesthetized with 10% pentobarbital sodium, they were fixed in the supine position on the small animal thermostatic pad on the operating table, and their fur was sheared, and their skins were prepared. Next, we performed endotracheal intubation (Zena, Shanghai, China) and mechanical ventilation by blind vision. Then, the skin of the rats' chest was cut, the thoracic tissue and aorta were separated, and the aortic arch was ligated by the insertion needle with 0.5 nonabsorbent suture (Zena, Shanghai, China) between the first and second branches. After taking out the pad and inserting the needle, we closed the chest cavity. The endotracheal intubation was removed when the rats fully resumed spontaneous breathing. Meanwhile, the sham group has only been performed thoracotomy, and the aorta was not ligated.

### 2.3. Experimental Animal Grouping

Experimental animals were divided into 3 groups: sham operation group (control group;*n* = 15; the rats underwent sham operation, and one week after the operation, they were intraperitoneally injected with normal saline (0.1 mL/100 g)); model group (*n* = 15; one week after aortic constriction, the rats were intraperitoneally injected with normal saline (0.1 mL/100 g)); and NAC treatment group (*n* = 15; one week after aortic constriction, rats were intraperitoneally injected with NAC (Tianpu, Guangzhou, China) (10 mg/kg/d), diluted with normal saline, and continuously administered for 2 weeks).

### 2.4. Echocardiography

Three weeks after the operation, MyLab30CV with a 10 MHz linear ultrasound transducer (Philips, Eindhoven, Netherlands) was used to complete the echocardiogram. The left ventricular diameter was measured on the parasternal long axis and short axis with a 50 Hz frame rate. The left ventricular end-diastolic diameter (LVEDd), left ventricular end-systolic diameter (LVESd), left ventricular ejection fraction (LVEF%), and left ventricular short-axis fractional shortening rate (LVFS%) were used to assess cardiac function.

### 2.5. Retention of Heart Tissue

The rats were anesthetized with intraperitoneal injection of 10% pentobarbital sodium 3 mL/kg. Then, the chest was opened along the midline incision to fully expose the heart, and blood was drawn at the root of the aorta and saved. The left ventricle was cut and weighed. Then, it was put in the refrigerator at -80°C. At the same time, the tibia of the rat was isolated and its length was measured.

### 2.6. Intracellular ROS Detection

The rat heart was immediately removed and placed in 1 mL of ice phosphate-buffered saline (PBS) liquid and shredded. Then, the cells were filtered with a 200-400 mesh screen, then centrifuged at 4°C, 1500 r/min for 5 minutes, and the supernatant was discarded. A cold PBS solution was added to prepare a single cell suspension, and the cell concentration was adjusted to 1 × 100-110 cells/mL. Then, 5 *μ*L 2′,7′-dichlorofluorescein yellow diacetate (DCFH-DA, Thermo Fisher Scientific, Waltham, MA, USA) was added and mixed well, then placed in a 37°C incubator in the dark for 30 minutes. It was centrifuged again, and the supernatant was discarded; 10% fetal bovine serum (FBS, Life Technology, Wuhan, China) was added, and the mixture was incubated at 37°C for 20 minutes. Finally, it was centrifuged at 1500 r/min for 5 minutes, and a single cell suspension of heart tissue was made. The cell suspension was sent to flow cytometry within 1 hour to detect the average fluorescence intensity of intracellular labeled fluorescent probes (Elabscience, Wuhan, China).

### 2.7. Masson Staining

The embedded heart tissue was cut into continuous 3-5 *μ*m thick slices with a microtome (Camilo Biological, Nanjing, China), and then the slides were dried in an oven at 55°C for 30 minutes. Slides were first dewaxed with xylene and then placed in gradient alcohol (100%~70%) for hydration. Then, the Masson kit (Walvax, Kunming, China) was used for dyeing. After the slides were dehydrated by gradient alcohol and xylene, the neutral resin was added dropwise and the coverslips were sealed. After the slides have dried, we observed and took pictures under the microscope (Elabscience, Wuhan, China).

### 2.8. Serological Test

Three weeks after the operation, 3 mL of right atrial blood was collected, centrifuged at 12,500 r/min for 10 minutes at low temperature to separate serum, and stored in a refrigerator at -80°C. According to the instructions provided by the reagent supplier (Jian Cheng, Nanjing, China), the glutathione (GSH), glutathione peroxidase (GPX), superoxide dismutase (SOD), lactic dehydrogenase (LDH) activity, and malondialdehyde (MDA) content in the serum of each group were separately detected.

### 2.9. Cell Extraction and Cell Culture

The heart of newborn SD rats was taken under sterile conditions, cut to about 1 mm^3^ small pieces, added with 1 g/L type II collagenase (Jian Cheng, Nanjing, China), stirred at 37°C with a constant temperature magnetic stirrer, and assisted in the separation of cells. After 5 minutes, the cells were collected, the digestion fluid was centrifuged at 1000 r/min for 5 minutes, and the pelleted cells were resuspended and filtered. The cells were cultured in a thermostatic 5% CO_2_ incubator at 37°C for 60 minutes using the differential attachment method. The supernatant was discarded, and the remaining cells were fibroblasts. Then, Dulbecco's Modified Eagle's Medium (DMEM, Life Technology, Wuhan, China) containing 10% FBS was added to continue the culture, and the medium was changed every 2-3 days. According to literature reports, 1 *μ*mol/L AngII (Tianpu, Guangzhou, China) was used to treat the second- to fourth-generation CFs for corresponding research.

### 2.10. MTT (3-(4,5-Dimethylthiazol-2-yl)-2,5-diphenyl Tetrazolium Bromide) Assay

CF cells in the logarithmic growth stage were seeded in 96-well plates with a density of 1 × 10^5^/mL. And cells were incubated in an incubator of 5% CO_2_ at 37°C. The cells were treated with 1 *μ*mol/L AngII for 24 hours and incubated at 37°C. After 24 hours, NAC was added to make the final concentration of NAC in each well 0, 10, 20, and 40 mmol/L, and the culture was continued to 48 hours. MTT (Yifei Xue, Nanjing, China) 20 mL of 5 mg/mL was added to each well. After the culture was continued for 4 hours, the culture medium was discarded, 150 *μ*L dimethyl sulfoxide (DMSO) was added to each well, and the plate shaker was shaken for 10 minutes. The absorbance was measured at 490 nm with an enzyme-linked immunoassay (Kaiji, Nanjing, China).

### 2.11. ^3^H Proline Admixture Method

The cells were cultured in a 96-well plate for 48 hours according to the above treatment, and ^3^H proline of 1 × 10^3^ Bq (Jian Cheng, Nanjing, China) and 50 mg/L ascorbic acid were added to each well, and the cells were further incubated for 4 hours. The number of living cells was examined by trypan blue staining. After the trypsin digested the cells into a cell suspension, the cells were collected by a cell collector onto a fiberglass filter paper. The fiberglass filter paper was placed in a scintillation counter tube, and each tube was filled with 0.5 mL of scintillation liquid. Then, we determined the radioactive intensity with a liquid scintillation counter (Jian Cheng, Nanjing, China), and the data obtained was expressed in cpm/well.

### 2.12. Western Blotting

Radioimmunoprecipitation assay (RIPA) protein lysate (Camilo Biological, Nanjing, China) was used to extract the cardiac tissue and CF total protein on ice, and the protein concentration of each group was detected and leveled by the bicinchoninic acid (BCA) method (Camilo Biological, Nanjing, China). Equal amounts of protein from each group were separated by sodium dodecyl sulfate-polyacrylamide gel electrophoresis (SDS-PAGE) and then transferred to the polyvinylidene difluoride (PVDF, Ye Sen, Shanghai, China) membrane. Then, the PVDF membrane was blocked with 5% skim milk at room temperature for 2 hours, and the primary antibody (GPX1, Abcam, Cambridge, MA, USA, 1 : 2000; GPX3, Abcam, Cambridge, MA, USA, 1 : 2000; NF-*κ*B, Abcam, Cambridge, MA, USA, 1 : 2000; I*κ*K-*α*, Abcam, Cambridge, MA, USA, 1 : 1000; I*κ*B-*α*, Abcam, Cambridge, MA, USA, 1 : 2000; Collagen I, Abcam, Cambridge, MA, USA, 1 : 2000; and GAPDH, Abcam, Cambridge, MA, USA, 1 : 5000) was added and incubated at 4°C overnight. The next day, after washing the PVDF membrane with PBS, the corresponding secondary antibody (Abcam, Cambridge, MA, USA, 1 : 1000) was added. After being incubated at room temperature for 2 hours, enhanced chemiluminescence (ECL, Yifei Xue, Nanjing, China) was added dropwise to expose in the gel imaging system.

### 2.13. RNA Isolation and Quantitative Real-Time Polymerase Chain Reaction (qRT-PCR)

A TRIzol reagent (Thermo Fisher Scientific, Waltham, MA, USA) was used to grind and extract the total RNA of rat heart tissues and CFs in each group on ice. After quantitative analysis, the equivalent amount of RNA was transcribed into complementary deoxyribonucleic acid (cDNA) using the reverse transcription kit (Thermo Fisher Scientific, Waltham, MA, USA). The same amount of cDNA was taken for PCR amplification. Finally, cDNA was detected according to the instructions of the fluorescence quantitative PCR kit (Thermo Fisher Scientific, Waltham, MA, USA). The relative expression level of the target gene was calculated by the 2^-*ΔΔ*Ct^ method. And the reaction conditions were as follows: 94°C denaturation 45 seconds, 59°C annealing 45 seconds, and 72°C extension 60 seconds, a total of 35 cycles. The primer sequence is shown in Table 1.

### 2.14. 5-Ethynyl-2′-deoxyuridine (EdU) Staining

Primary fibroblasts were cultured with a diluted EdU reagent (Kaiji, Nanjing, China). The next day, PBS was used to wash the cells, and after fixation, Triton X-100 (Kaiji, Nanjing, China) was used to break the membrane and 5% goat serum was used to seal it. Then, the reaction solution was prepared according to the kit instructions and incubated for half an hour at room temperature. After the cells were rinsed with PBS three times, a DAPI reagent (Thermo Fisher Scientific, Waltham, MA, USA) was used to stain the nuclei. Finally, the cells were observed and photographed under an inverted fluorescence microscope.

### 2.15. Statistical Analysis

Statistical Product and Service Solutions (SPSS) 20.0 software (IBM, Armonk, NY, USA) was used for statistical analysis. The experimental data were expressed as mean ± standard deviation ($\bar{X}\pm \text{SD}$). Differences between the two groups were analyzed by using Student's *t*-test. Comparison between multiple groups was done using the one-way ANOVA test followed by the post hoc test (least significant difference). *P* < 0.05 was considered statistically significant.

## 3. Results

### 3.1. NAC Improves the Damaged Heart Structure of Rats after Aortic Constriction

**Figure 1:**
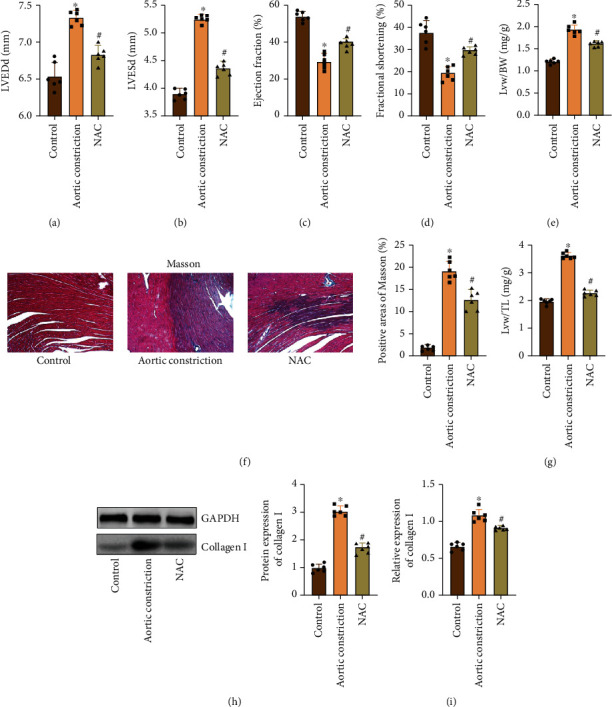
NAC improves the damaged heart structure of rats after aortic constriction. (a) Echocardiography detected the LVEDd of rats. (b) Echocardiography detected the LVESd of rats. (c) Echocardiography detected the LVEF% of rats. (d) Echocardiography detected the LVFS% of rats. (e) Ratio of Lvw/BW. (f) Ratio of Lvw/TL. (g) Masson staining and positive area analysis (magnification: 200x). (h) WB detected the Collagen I expression and gray value analysis. GAPDH was used as an internal control. (i) qRT-PCR detected the Collagen I expression. “∗” indicates the statistical difference from the control group, *P* < 0.05; “#” indicates the statistical difference from the aortic constriction group, *P* < 0.05. LVEDd: left ventricular end-diastolic diameter; LVESd: left ventricular end-systolic diameter; LVEF: left ventricular ejection fraction; LVFS: left ventricular fractional shortening; Lvw: left ventricular weight; BW: body weight; TL: tibial length.

To determine whether NAC protects the heart from cardiac impairment caused by overloading, the results of echocardiography showed that the diameter of ventricular dilatation of the model group was obviously higher than that of the control group, which showed that LVEDd and LVESd were obviously increased. In addition, the cardiac contractile function was obviously lower than that of the control group, which showed that the LVEF% and the LVFS% were reduced. In the NAC treatment group, the diameter of ventricular dilatation was lower than that of the model group, which showed that LVEDd and LVESd were obviously lower than those of the model group. The systolic function of the heart was obviously higher than that of the model group, showing that the LVEF% and the LVFS% were increased compared with those of the model group (Figures 1(a)–1(d)). At the same time, we detected the ratio of Lvw/HW and Lvw/TL, and we found that the Lvw/HW and Lvw/TL values of the model group were obviously higher than those of the control group, but the Lvw/HW and Lvw/TL values of the NAC group were lower than those of the model group (Figures 1(e) and 1(f)). Then, Masson staining found that the myocardial tissue structure of the control group was normal and the fibrosis was not obvious, while the myocardial tissue structure of the model group was disordered and the fibrosis was obvious. Conversely, the myocardial tissue structure of the rats in the NAC treatment group was slightly disordered and the fibrosis area was relatively reduced (Figure 1(g)). Next, we detected the expression of Collagen I by WB and qRT-PCR and found that the expression of Collagen I in the model group was the highest, while the expression of Collagen I in the NAC group was lower than that in the model group (Figures 1(h) and 1(i)).

### 3.2. NAC Improves the Damaged Heart Function of Rats after Aortic Constriction

**Figure 2:**
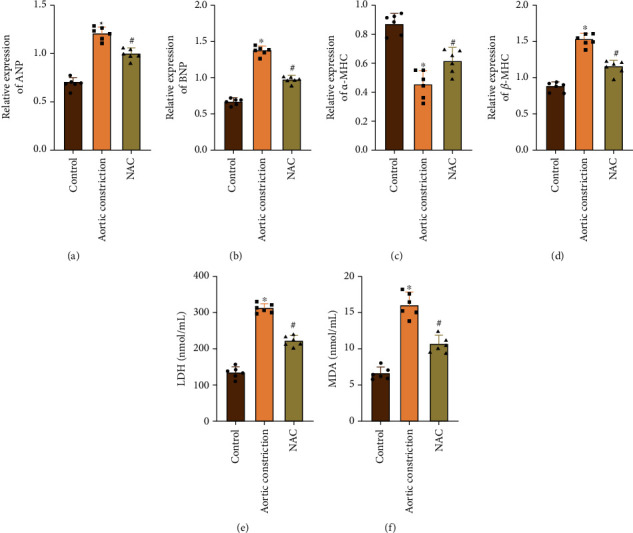
NAC improves the damaged heart function of rats after aortic constriction. (a) qRT-PCR detected the ANP expression. (b) qRT-PCR detected the BNP expression. (c) qRT-PCR detected the *α*-MHC expression. (d) qRT-PCR detected the *β*-MHC expression. (e) The kit detected the serum LDH activity. (f) The kit detected the serum MDA content. “∗” indicates the statistical difference from the control group, *P* < 0.05; “#” indicates the statistical difference from the aortic constriction group, *P* < 0.05. ANP: atrial natriuretic peptide; BNP: B-type natriuretic peptide; *α*-MHC: *α*-myosin heavy chain; *β*-MHC: *β*-myosin heavy chain; LDH: lactic dehydrogenase; MDA: malondialdehyde.

Firstly, qRT-PCR results showed that, compared with the control group, model group rats' cardiac tissue hypertrophy-related gene has obvious differences with that of the control group, including atrial natriuretic peptide (ANP), B-type natriuretic peptide (BNP), and *β*-myosin heavy chain (*β*-MHC) transcriptional expressions, which were higher than those of the control group, and *α*-myosin heavy chain (*α*-MHC) transcriptional expression was lower than that of the control group. In contrast, transcriptional expressions of the NAC group, ANP, BNP, and *β*-MHC, were lower than those of the model group, while transcriptional expression of *α*-MHC was higher than that of the model group (Figures 2(a)–2(d)). At the same time, we found through serological detection that the LDH activity and MDA content in the model group obviously increased, while the LDH activity and MDA content obviously decreased after NAC treatment (Figures 2(e) and 2(f)).

### 3.3. NAC Improves Oxidative Stress Response after Aortic Constriction

**Figure 3:**
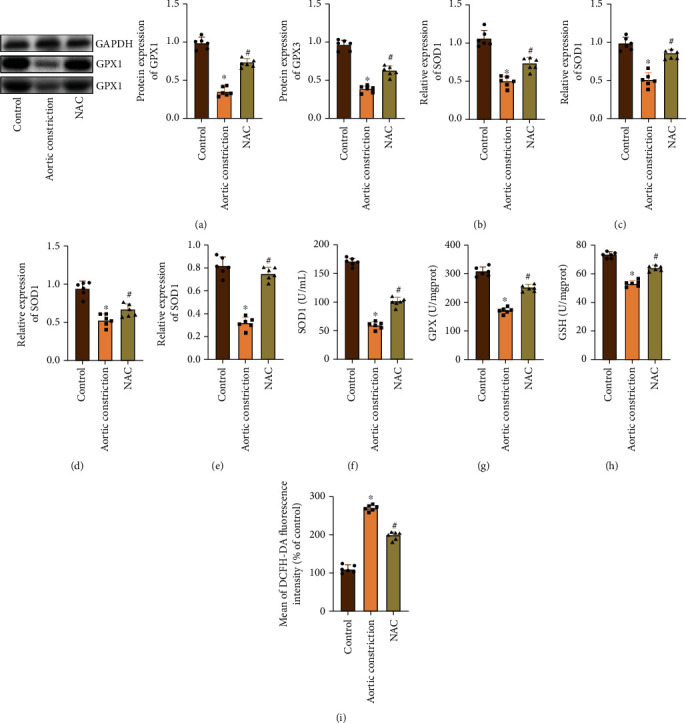
NAC improves oxidative stress response after aortic constriction. (a) WB detected the expression of GPX1 and GPX3 and gray value analysis. GAPDH was used as an internal control. (b) qRT-PCR detected the expression of SOD1. (c) qRT-PCR detected the expression of SOD2. (d) qRT-PCR detected the expression of GPX1. (e) qRT-PCR detected the expression of GPX3. (f) The kit detected the serum SOD activity. (g) The kit detected the serum GPX activity. (h) The kit detected the serum GSH activity. (i) Flow cytometry detected the intracellular ROS levels. “∗” indicates the statistical difference from the control group, *P* < 0.05; “#” indicates the statistical difference from the aortic constriction group, *P* < 0.05. SOD: superoxide dismutase; GPX: glutathione peroxidase; GSH: glutathione; ROS: reactive oxygen species.

Firstly, we detected the expression of GPX1 and GPX3 proteins in the myocardial tissues of each group by WB (Figure 3(a)). The results showed that the expression of GPX1 and GPX3 was obviously decreased in the model group, while NAC effectively inhibited the expression of GPX1 and GPX3 after treatment. At the same time, qRT-PCR detection of SOD1 mRNA, SOD2 mRNA, GPX1 mRNA, and GPX3 mRNA also found similar results; NAC treatment can reverse the decreased transcriptional expression of SOD1, SOD2, GPX1, and GPX3 in myocardial tissue (Figures 3(b)–3(e)). Then, we found that the activities of SOD, GPX, and GSH in the model group were obviously reduced by serological detection. On the contrary, we found that the expression of SOD, GPX, and GSH in the NAC group was higher than that in the model group (Figures 3(f)–3(h)). In addition, flow cytometry detection of ROS levels in myocardial tissue revealed that ROS levels were obviously increased in the model group, while NAC can promote ROS clearance (Figure 3(i)).

### 3.4. NAC Inhibits the NF-*κ*B Signaling Pathway

**Figure 4:**
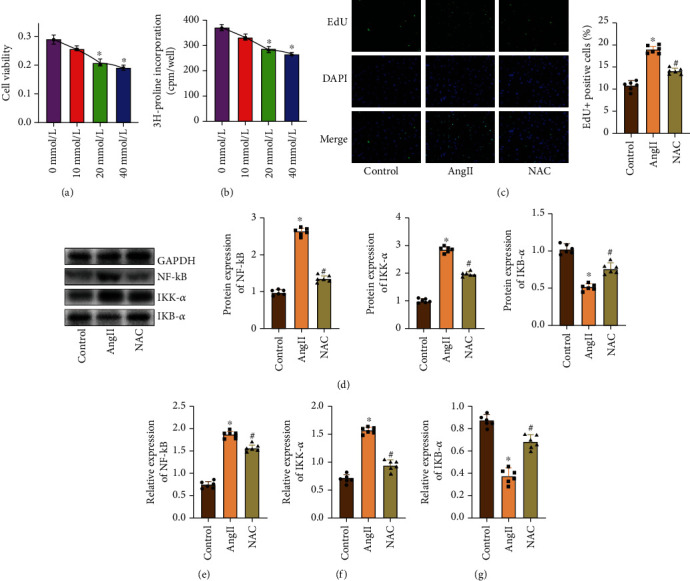
NAC inhibits the NF-*κ*B signaling pathway. (a) MTT detected the CF activity. (b) The ^3^H proline incorporation method detected the cell total collagen synthesis function. (c) EdU staining detected the cell proliferation and percentage analysis of positive cells. (d) WB detected the expression of NF-*κ*B, I*κ*K-*α*, I*κ*B-*α*, and gray value analysis. GAPDH is used as an internal control. (e) qRT-PCR detected the expression of NF-*κ*B. (f) qRT-PCR detected the expression of I*κ*K-*α*. (g) qRT-PCR detected the expression of I*κ*B-*α*. “∗” indicates the statistical difference from the control group, *P* < 0.05; “#” indicates the statistical difference from the aortic constriction group, *P* < 0.05.

In order to detect whether NAC inhibits CF proliferation and collagen synthesis through the NF-*κ*B signaling pathway, we investigated the effect of NAC *in vitro* by AngII treatment of CFs. First of all, the results of the MTT experiment showed that compared with the control group, NAC of 10, 20, and 40 mmol/L had an inhibitory effect on the growth of CF cells, and there were differences among groups with different concentrations (Figure 4(a)). Also, we found that when NAC concentration was at 20 mmol/L, its inhibition activity of CFs was significant. Therefore, in our subsequent experiments, we chose this concentration to treat CFs [14]. At the same time, the results of the ^3^H proline incorporation method showed that compared with the control group, the synthesis of total collagen of CFs decreased obviously after NAC treatment for 48 hours, and the amount of doping also decreased with the increase of NAC concentration (Figure 4(b)). Then, we detected the proliferation of CFs by EdU staining, and the results showed that NAC treatment could significantly inhibit the proliferation of CFs induced by AngII (Figure 4(c)). In addition, WB detection found that after AngII treated CFs, the NF-*κ*B signaling pathway was activated, while NAC treatment can increase the levels of I*κ*B-*α* protein and decrease the levels of NF-*κ*B and I*κ*K-*α* protein (Figure 4(d)). At the same time, qRT-PCR also produced similar results (Figures 4(e)–4(g)).

## 4. Discussion

The remodeling of the heart includes myocardial parenchymal remodeling and interstitial remodeling. Parenchymal remodeling is mainly caused by pathological hypertrophy or degeneration and necrosis of cardiomyocytes, while interstitial remodeling includes activation and proliferation of fibroblasts and changes in the composition and amount of the extracellular matrix collagen network [15]. Therefore, in the process of CR, CFs and the extracellular matrix proliferate and infiltrate a lot, leading to heart fibrosis. This process is an important step in the change of heart function from decompensation to HF. Collagen I and Collagen III are secreted by CFs and are the main components of the extracellular matrix of the heart. In the process of CR, pathological hyperplasia of Collagen I and Collagen III caused an imbalance of the myocardial parenchymal and interstitial tissues, and the number of myocardial cells as working cells decreased relatively, which generally showed the body's cardiac dysfunction [16]. Our results also found that in the model group, the cardiac tissue structure was disordered, and the fibrosis area and Collagen I expression were obviously higher than those in the control group. At the same time, the heart function of rats in the model group decreased, and some hypertrophy-related indicators were obviously increased. Thus, we speculate that rational remodeling of heart disease has occurred in rats.

In the cardiovascular system, NAC has also received attention, and it can play a beneficial role in the body under the conditions of coronary atherosclerosis, myocardial IRI, and hypertension and CR [17, 18]. Research studies have found that the plasma TNF-*α* concentration in the rat model of hypertension was obviously associated with a decrease in cardiac LVFS% and a lack of cardiac GSH. However, after NAC intervention, there was no effect on hypertension, but the plasma TNF-*α* concentration reached normal levels, increased LVFS% and restricted the left ventricular posterior wall hypertrophy, and inhibited the activation of matrix metalloproteinase 2 and matrix metalloproteinase 9, which reduces the collagen deposition in the left ventricle, suggesting that NAC can slow down the process of CR in hypertensive rats and effectively protect cardiac function [19]. In addition, there are research studies that believe that NAC may directly prevent blood pressure, promote the synthesis of GSH in the heart to inhibit the harmful effects of inflammatory factors on the heart, or directly inhibit the activation of NF-*κ*B through GSH and other mechanisms. And it can reverse the effects of hypertensive CR [20]. However, there is less relevant evidence, and its mechanism still needs further study, especially the direct effect of fibroblasts.

Studies in other systemic diseases have shown that NAC can directly affect the proliferation and collagen synthesis of mouse and human lung fibroblasts, reduce the expression of cyclin E, block cell proliferation in the G1 phase, and thus inhibit cell proliferation, and it can reduce TGF-*α*-induced or non-TGF-*α*-induced collagen synthesis [21]. The results of this experiment indicated that NAC had inhibitory effects on proliferation and collagen synthesis of rat CFs similar to those mentioned in the previous experiment, but the specific mechanism of NAC and its effect on overall cardiac fibrosis under pathological conditions still need to be further studied.

Cell pathways activated by OS led to programmed cell death, which eventually leads to cardiac dysfunction and HF. It can be seen that the signal transduction pathway mediated by OS is particularly important in the formation of MH [22, 23]. Therefore, we speculate that MF is closely related to the OS pathway. NF-*κ*B is a family of transcription factor proteins. And it is very sensitive to ROS produced by OS. Activated NF-*κ*B can cause cells to produce numerous cytokines, and research studies have shown that ROS can lead to the activation of I*κ*K-*α*, thereby promoting the activation of NF-*κ*B [24, 25]. Our results also found that *in vivo*, with the model group heart tissue redox imbalance, antioxidant enzyme activity decreased, prompting the accumulation of ROS in cells. *In vitro*, AngII treated CFs and induced the activation of the NF-*κ*B signaling pathway. NAC can restore antioxidant enzyme activity in the body and remove excess ROS in cells. At the same time, NAC can inhibit the total collagen synthesis function of cells and inhibit the excessive activation of the NF-*κ*B signaling pathway *in vitro*. Although the preliminary research results of this experiment suggest that NAC can be considered a potential treatment method for rational reconstruction of heart disease, it is necessary to further study the comprehensive impact of all aspects before it can provide a stronger basis for treatment.

Our study confirmed that *in vivo*, antioxidant enzyme activity and intracellular ROS content are closely related to myocardial remodeling, and NAC can significantly relieve OS *in vivo*, thus inhibiting MF and remodeling. Meanwhile, *in vitro*, we first reported that NAC inhibits CF proliferation and collagen synthesis through the NF-*κ*B pathway, thus providing a new research basis for the treatment of MF and remodeling.

## 5. Conclusions

NAC can inhibit AngII-induced CF proliferation and collagen synthesis through the NF-*κ*B signaling pathway, alleviate the OS response of myocardial tissue, inhibit the fibrosis of myocardial tissue, and thus slow down the pathological remodeling of the heart. This also provides a new research basis for the treatment of CR.

## Figures and Tables

**Table 1:** Real-time PCR primers.

Gene name	Forward (5′ > 3′)	Reverse (5′ > 3′)
*α*-MHC	GCCCAGTACCTCCGAAAGTC	GCCTTAACATACTCCTCCTTGTC
*β*-MHC	ACTGTCAACACTAAGAGGGTCA	TTGGATGATTTGATCTTCCAGGG
Sirt1	CCAGATCCTCAAGCCATG	TTGGATTCCTGCAACCTG
SOD1	GGTGAACCAGTTGTGTTGTC	CCGTCCTTTCCAGCAGTC
SOD2	CAGACCTGCCTTACGACTATGG	CTCGGTGGCGTTGAGATTGTT
GPX1	ATCATATGTGTGCTGCTCGGCTAGC	TACTCGAGGGCACAGCTGGGCCCTTGAG
GPX3	AGAGCCGGGGACAAGAGAA	ATTTGCCAGCATACTGCTTGA
ANP	GCTTCCAGGCCATATTGGAG	GGGGGCATGACCTCATCTT
BNP	GAGGTCACTCCTATCCTCTGG	GCCATTTCCTCCGACTTTTCTC
I*κ*K-*α*	GTCAGGACCGTGTTCTCAAGG	GCTTCTTTGATGTTACTGAGGGC
I*κ*B-*α*	GGATCTAGCAGCTACGTACG	TTAGGACCTGACGTAACACG
P65	ACTGCCGGGATGGCTACTAT	TCTGGATTCGCTGGCTAATGG
GAPDH	ACAACTTTGGTATCGTGGAAGG	GCCATCACGCCACAGTTTC

qRT-PCR: quantitative real-time polymerase chain reaction.

## Data Availability

The datasets used and analyzed during the current study are available from the corresponding author on reasonable request.
